# Symmetric Electrodes for Electrochemical Energy‐Storage Devices

**DOI:** 10.1002/advs.201600115

**Published:** 2016-06-08

**Authors:** Lei Zhang, Shi Xue Dou, Hua Kun Liu, Yunhui Huang, Xianluo Hu

**Affiliations:** ^1^ State Key Laboratory of Materials Processing and Die and Mould TechnologySchool of Materials Science and EngineeringHuazhong University of Science and TechnologyWuhan430074P. R. China; ^2^ Institute for Superconducting and Electronic MaterialsUniversity of WollongongWollongongNSW2522Australia

**Keywords:** electrochemical energy storage, lithium‐ion batteries, sodium‐ion batteries, supercapacitors, symmetric electrodes

## Abstract

Increasing environmental problems and energy challenges have so far attracted urgent demand for developing green and efficient energy‐storage systems. Among various energy‐storage technologies, sodium‐ion batteries (SIBs), electrochemical capacitors (ECs) and especially the already commercialized lithium‐ion batteries (LIBs) are playing very important roles in the portable electronic devices or the next‐generation electric vehicles. Therefore, the research for finding new electrode materials with reduced cost, improved safety, and high‐energy density in these energy storage systems has been an important way to satisfy the ever‐growing demands. Symmetric electrodes have recently become a research focus because they employ the same active materials as both the cathode and anode in the same energy‐storage system, leading to the reduced manufacturing cost and simplified fabrication process. Most importantly, this feature also endows the symmetric energy‐storage system with improved safety, longer lifetime, and ability of charging in both directions. In this Progress Report, we provide the comprehensive summary and comment on different symmetric electrodes and focus on the research about the applications of symmetric electrodes in different energy‐storage systems, such as the above mentioned SIBs, ECs and LIBs. Further considerations on the possibility of mass production have also been presented.

## 1 Introduction

Global concerns over depleting fossil fuels require us to move away from fossil energy towards the area of renewable energy. As a result, the electrical energy‐storage systems become a research focus in energy conversion obtained from renewable resources, such as solar and wind power, to energy grid to store the intermittent renewable energy.^1^, ^2^, ^3^, ^4^, ^5^, ^6^, ^7^, ^8^, ^9^, ^10^, ^11^, ^12^ Rechargeable batteries and supercapacitor are some typical ones among such energy‐storage systems, and are playing vital roles in portable electronics.^13^, ^14^, ^15^, ^16^, ^17^, ^18^, ^19^, ^20^, ^21^ Nowadays, the existing rechargeable energy‐storage systems include sodium‐ion batteries (SIBs), lithium‐ion batteries (LIBs), and supercapacitors. Owing to some intrinsic disadvantages of the employed electrodes, the practical utilization of these systems is still hindered by the high safety risk, sophisticated production process and high cost.^22^, ^23^, ^24^, ^25^, ^26^, ^27^, ^28^, ^29^ Therefore, the search for new electrode materials with scalable production, low cost, high energy density, and high safety has attracted tremendous attention.

Symmetric electrodes, as the name implies, are two identical electrodes that can be employed as both the cathode and anode in an individual energy‐storage system.^30^ Apart from the electrochemical energy‐storage systems, other kinds of fuel cells can also be assembled with symmetric electrodes, such as the Zr‐doped SrFeO_3−_ *_δ_* based symmetric fuel cell.^31^ Before the symmetric batteries are assembled, such as symmetric LIBs, one electrode must be in a lithiated state, while the other is in a delithiated state. Therefore, only the electrode potentials present in the cell can be tested, which means a large potential difference between the lithiated and delithiated symmetric electrodes is necessary. In addition, the structure of these symmetric electrodes should be kept stable even under a high or low working potential. Similar to a full cell, only limited amount of lithium can be supplied and transferred between electrodes during cycling. As a result, any parasitic reactions in the cell can lead to a capacity loss due to the absence of the lithium or sodium foil. Owing to such a unique configuration, however, the cell may be overcharged to some extent and can buffer the large volume expansion (cathode expansion accompanied by anode shrinking, and vice versa) more effectively. Moreover, the manufacturing costs and the fabrication process can be greatly reduced and simplified in terms of commercial viewpoints. The processing cost of the electrode coating can be reduced, and only one type of the electrode needs to be produced, which makes it more attractive for the scalable production of next‐generation energy‐storage systems. In addition, the use of symmetric electrodes may eliminate the possibility of side reactions between the electrolyte (or electrolyte additives) and the lithium (or sodium) foil. Those side reactions could be restricted if the same electrolyte is used in a full cell, improving the safety and cyclability.^30^ In general, an in‐time review on the recent progress of symmetric electrodes in different symmetric electrochemical energy‐storage systems could inspire the rational design of novel high‐performance electrodes in a variety of energy‐storage devices. Furthermore, from a scientific perspective, the investigation on the identical electrodes that work during different potential ranges in one system is of particular interest.

In this Progress Report, we aim to provide a comprehensive summary and comments on different symmetric electrodes in different energy‐storage systems, which may help shed light on the newly emerging energy‐storage techniques.

## 2 Symmetric Electrodes in Different Energy‐Storage Systems

### 2.1 Symmetric LIBs

Among all the various available energy‐storage technologies, LIBs play a dominant role in portable electronic devices and have become the prime candidate to power the next‐generation electric vehicles and plug‐in hybrid electric vehicles.^32^, ^33^, ^34^, ^35^, ^36^ At present, the commercial anodes in LIBs are mainly carbon‐based and silicon‐based materials. Unfortunately, the theoretical specific capacity of carbon‐based anodes (≈372 mAh g^–1^) is low, and the volume expansion (over 400% in the fully lithiated state) of silicon‐based anodes is large. They cannot satisfy the ever‐increasing demand for high energy.^37^, ^38^, ^39^, ^40^ In addition, the high risk resulted from the combustible nature of organic electrolytes and side reactions between the electrolyte and electrodes is a significant challenge for LIBs.^41^ As a result, manufacturing of LIBs with symmetric electrodes would be an effective way to improve the overall performance.

#### 2.1.1 Phosphate Salts‐Based Symmetric LIBs

NASICON‐type lithium vanadium phosphate, Li_3_V_2_(PO_4_)_3_ (LVP), has been identified as electroactive cathode materials for LIB applications because of its high theoretical capacity of 197 mAh g^−1^ and unique crystal structure that enables three‐dimensional (3D) pathways for Li^+^ insertion/extraction.^42^, ^43^, ^44^, ^45^ The insertion and extraction reactions of Li^+^ ions in monoclinic LVP electrodes occur at around 4.1 and 1.7 V, corresponding to the redox reactions of V^+4^/V^+3^ and V^+3^/V^+2^, respectively. Such intriguing properties make the monoclinic LVP attractive as both an anode and a cathode in the LIBs.

All‐solid‐state phosphate symmetric LIBs using LVP as both the cathode and the anode were proposed by Okada's group.^46^ This symmetric cell exhibited an output voltage of 2 V based on the redox reactions of V^+4^/V^+3^ and V^+3^/V^+2^ at about 2 and 4 V. Also, the discharge capacities of 104 mAh g^−1^ at 2 μA cm^−2^ at 80 °C and 42 mAh g^−1^ at 25 °C were obtained.

**Figure 1:**
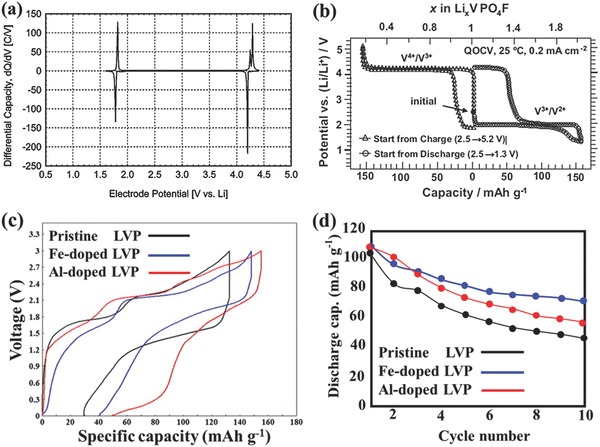
a) Electrochemical voltage spectroscopy (EVS) data for a typical Li‖LiVPO_4_F cell cycled between 1.60–4.40 V. The data shown were collected at 23 °C and are for the first cycle. The electrolyte comprised a 1 m LiPF_6_ solution in ethylene carbonate/dimethyl carbonate (2:1 by weight). Reproduced with permission.^43^ Copyright 2005, The Electrochemical Society. b) QOCV curves for typical half‐cell Li/[1m] LiPF_6_/EC‐DMC (1:1)/LiVPO_4_F cycled in two potential ranges from 2.5 to 5.2 V beginning with charging and from 2.5 to 1.3 V beginning with discharging. The data were collected at 0.2 mA cm^−2^ and room temperature. Reproduced with permission.^47^ Copyright 2010, Elsevier. c) Initial charge and discharge curves of pristine Li_3_V_2_(PO_4_)_3_, Fe‐doped Li_3_V_2_(PO_4_)_3_, and Al‐doped Li_3_V_2_(PO_4_)_3_ of all‐solid‐state symmetric batteries at a rate of 7.5 mA g^−1^ in the voltage range of 0–3.0 V with Li_1.5_Al_0.5_Ge_1.5_(PO_4_)_3_ solid‐state electrolyte, and d) their cyclabilities. Reproduced with permission.^56^ Copyright 2015, Elsevier.

In order to improve the thermal stability of LIBs, Yamaki's group^47^ proposed the room‐temperature molten salts of LiBF_4_/1‐ethyl‐3‐methyl imidazolium tetrafluoroborate (EMIBF_4_) as the ionic liquid (IL) electrolyte in a LVP‐based symmetric LIB. They found that the IL‐based symmetric cells revealed better cyclability and a more stable behavior at elevated temperatures (80 °C). In addition, electrochemical properties of lithium vanadium fluorophosphates (LiVPO_4_F) have been investigated in detail by Barker and co‐workers.^48^ LiVPO_4_F was endowed a V^3+^/V^4+^ redox couple at around 4.2 V vs Li/Li^+^, which is about 0.3 V higher than that of the pure LVP, due to the introduction of the electronegative F atom. Apart from the higher operating voltage, an additional Li^+^ reversible reaction at around 1.8 V associated with the V^2+^/V^3+^ redox couple was also found in this composite. These advantages allow LiVPO_4_F more promising as the symmetric electrode in LIBs. From this point of view, Bryan's group described the preliminary electrochemical performance of a novel LiVPO_4_F‐based symmetric LIB (see **Figure** **1**a).^43^ This symmetric cell offered a relatively flat discharge profile, with an average discharge voltage of 2.4 V. In 2011, Yamaki's group also reported the fabrication of a LiVPO_4_F//LiVPO_4_F symmetric LIB using an IL‐based electrolyte.^47^ Based on the quasi‐open circuit voltage (QOCV) result (see Figure 1b), an optimal charge cut off voltage at about 2.8 V and an initial Coulombic efficiency of as high as 93% were achieved.

Besides the F doping in LVP, other kinds of metal ions (such as Mg,^49^ Ti,^50^ Fe,^51^ Co,^52^, ^53^ Ni,^54^ Mn,^55^ and Al^56^) were also good candidates to improve the electrochemical performance of LVP. Among those doped LVPs, the electrode of Li_3_V_1.9_M_0.1_(PO_4_)_3_ (M = Fe, Al) was first employed in an all‐solid‐state symmetric LIBs by Okada's team.^56^ Using the solid‐state electrolyte of Li_1.5_Al_0.5_Ge_1.5_(PO_4_)_3_, the Li_3_V_1.9_Fe_0.1_(PO_4_)_3_ and Li_3_V_1.9_Al_0.1_(PO_4_)_3_ electrodes showed initial discharge capacities of 107.6 and 105.6 mAh g^−1^ at a rate of 7.5 mA g^−1^, respectively. The Fe‐doped sample exhibited the best cycling stability (see Figure 1c and 1d). The authors explained that the enhanced discharge capacities of Fe and Al‐doped LVP could be attributed to the increased electrical conductivity induced by the introduction of Fe^3+^ or Al^3+^ trivalent ions into the V^3+^ sites.

In summary, the electrochemical performance of the phosphate‐based electrodes with different doped elements can be improved significantly, compared with the pure phosphate‐based symmetric electrodes. This mainly benefits from the increased conductivity, structural stability and output potential arising from the introduction of extra elements.

#### 2.1.2 Titanate Salts‐Based Symmetric LIBs

Apart from the practical applications in the energy‐storage field, symmetric cells are good candidates for exploring the electrodes, electrolytes, and electrolyte additives in only a limited potential range of the electrode in an energy‐storage device, rather than the devices made of both a high‐potential positive electrode and a low‐potential negative electrode in a full lithium‐ion cell.^30^ Taking advantages of the symmetric cell, fundamental studies on the effects of electrolyte additives and new electrode materials on the cell degradation could be well conducted. Based on a lithium titanate/lithium titanate symmetric cell, Dahn's group found that the Coulombic efficiency could be accurately calculated by using a standard charger that was able to measure capacity fade well.^30^ Moreover, based on our review about the titanate‐based symmetric electrodes in SIBs which will be mentioned and discussed in the following part, we believe that titanates should also be promising candidates as symmetric electrodes for LIBs.

#### 2.1.3 Vanadate Salts‐Based Symmetric LIBs

**Figure 2:**
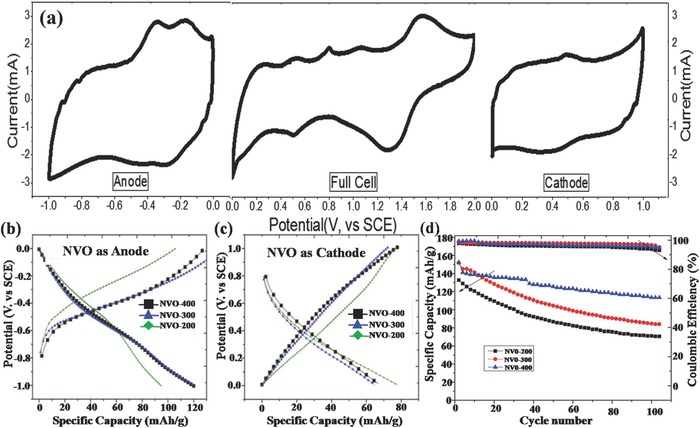
a) Cyclic voltammograms (CV) of NVO‐400 as cathode, full cell, anode in three‐electrode configuration (vs SCE) from −1.0 to 0 V, 0 to 2.0 V, and 0 to 1.0 V respectively, at scan rate of 5 mV s^−1^, galvanostatic charge–discharge profiles of NVO‐200, NVO‐300 and NVO‐400, respectively. b) Anode in three‐electrode configuration (vs SCE) from −1 to 0 V. c) cathode in three‐electrode configuration (vs SCE) from 0 to 1 V, in 4 m aqueous solution of LiCl, at a current density of 5 A g^−1^. d) specific capacity vs cycle number and Coulombic efficiency of NVO‐200/NVO‐200, NVO‐300/NVO‐300 and NVO‐400/NVO‐400, respectively, in three‐electrode configuration from 0 to 1.9 V, at current density of 5 A g^−1^. Reproduced with permission.^41^ Copyright 2014, The Electrochemical Society.

The layered lithium trivanadate (LiV_3_O_8_) cathode material has attracted considerable attention because of its low cost, high specific capacity (about 200 mAh g^–1^), and good safety features.^57^ However, the commercial application of this composite suffers from its poor cycling stability in an aqueous electrolyte, as a result of the collapse‐prone crystal structure and the dissolution of vanadium during cycling.^58^, ^59^ One way to address this problem is to replace Li^+^ with Na^+^ in LiV_3_O_8_, leading to a structure of sodium vanadate (Na_1.16_V_3_O_8_, NVO). It is more tolerant to lithium intercalaion/deintercalation with a less vanadium‐dissolution degree, in contrast to LiV_3_O_8_.^41^ Meanwhile, taking the wide operating potential window of NVO into consideration, it is interesting to assemble a symmetric LIB based on the NVO electrodes. Recently, Madhavi's group first reported the applicability of Na_1.16_V_3_O_8_ as both positive and negative electrodes, achieving a symmetric Na_1.16_V_3_O_8_‐based aqueous rechargeable LIB.^41^ The electrochemical performances of the NVO samples prepared by thermal treatment at different temperatures of 200 °C (Sample NVO‐200), 300 °C (Sample NVO‐300), and 400 °C (Sample NVO‐400) were explored in detail. Among them, Sample NVO‐400 exhibited a higher initial capacity of more than 150 mAh g^−1^ at a high current rate and retained around 75% of the initial capacity after 100 cycles, demonstrating the superior rate capability and cyclability of the NVO‐based symmetric electrodes (**Figure** **2**). Compared with the commercial full LIBs, the energy densities of these symmetric LIBs are relatively lower due to the narrower operating potential. Nevertheless, other merits like high safety would make it suitable for some special applications where considerations such as cost, ease of construction, and safety are at a premium.

### 2.2 Symmetric SIBs

The device of LIBs is one of the most successful energy‐storage systems at present. Nevertheless, the limited reserve of lithium on the earth is bringing about the increasing cost for the scalable production of LIBs and a widespread awareness for finding the new alternative energy‐storage technology. Very recently, tremendous attention has been paid to the SIBs because of the abundant sodium sources and the higher value of the Na/Na^+^ potential (–2.7 V versus standard hydrogen electrode (SHE)) compared to Li/Li^+^ (–3.0 V versus SHE). This can effectively reduce the electrolyte degradation at the surface of the electrode material. During the past 6 years, the symmetric SIB as a new energy‐storage system was attracting more and more attention. Similar to LIBs, phosphate and titanate‐based materials are also good candidates as the symmetric electrodes in SIBs.

#### 2.2.1 Phosphate Salts‐Based SIBs

Na_3_V_2_(PO_4_)_3_ (NVP), a typical NASICON‐related phosphate, is an attractive candidate for active electrochemical material in SIBs. Since the electric potential between the two redox couples of V^4+^/V^3+^ and V^3+^/V^2+^ is large (similar to LVP in symmetric LIBs), NVP has showed its great potential in symmetric SIBs. In 2010, Yamaki's group fabricated NVP‐based symmetric SIBs. It was found that this symmetric electrode had a better electrochemical performance in IL electrolytes, compared with the organic electrolytes.^60^ In order to further improve the safety of this symmetric SIB, Yamaki's group also reported a new type all‐solid‐state NVP‐based symmetric cell, employing the Na_3_Zr_2_Si_2_PO_12_ as the electrolyte.^61^ It was found that this cell could work as a secondary battery and showed initial discharge capacities of 68 and 32 mAh g^−1^ at the current densities of 1.2 and 10 μA cm^–2^, respectively. Unfortunately, like most polyanion systems, the slightly distorted and separated VO_6_ octahedra minimize the electronic conductivity of the compound, which brings down its performance. Therefore, the conductivity and cyclability of the pure NVP need to be enhanced. Among various approaches, carbon matrices have a significant influence on the conductivity to improve the electrochemical performance of NVP. Therefore, the effects of different carbon matrices including acetylene carbon (AC) nanospheres, carbon nanotubes (CNTs), and graphite nanosheets on the active NVP are investigated, and a reasonable mechanism was proposed by Mai and co‐workers.^32^ They also found that NVP distributed in the AC nanospheres has the best SIB performance (approximately 100% of the theoretical capacity at 0.5 C and 96.4% capacity retention at 5 C after 200 cycles). In contrast, the performance of NVP in the CNT matrix is moderate, and the performance of NVP in the graphite nanosheets is inferior.

#### 2.2.2 Titanate Salts‐Based SIBs

**Figure 3:**
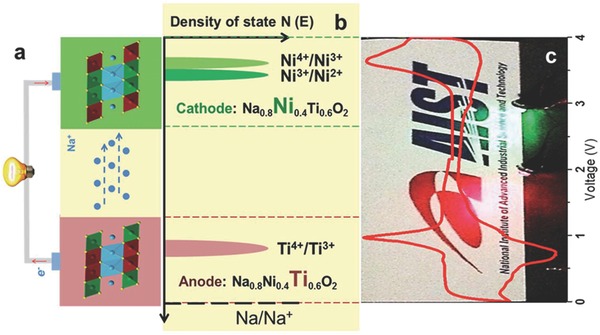
Schematic illustration of the symmetric SIB design via the two redox couples of nickel and titanium in the layered material Na_0.8_Ni_0.4_Ti_0.6_O_2_. a) A diagram of the proposed symmetric cell based on O3‐type Na_0.8_Ni_0.4_Ti_0.6_O_2_. b) Schematic of energy vs density of states plot, showing the relative positions of the Fermi energy level of Ni^4+^/Ni^2+^ (Ni^4+^/Ni^3+^ and Ni^3+^/Ni^2+^) and Ti^4+^/Ti^3+^ redox couples for O3‐type Na_0.8_Ni_0.4_Ti_0.6_O_2_. c) The CV curve of Na_0.8_Ni_0.4_Ti_0.6_O_2_/Na half‐cell in the whole voltage range of 0–4 V vs Na^+^/Na; the background shows the lighted LED bulbs driven by the designed bipolar, Na_0.8_Ni_0.4_Ti_0.6_O_2_‐based symmetric cells. Reproduced with permission.^64^ Copyright 2015, The Royal Society of Chemistry.

Layered nickelate and titanate‐based composites, such as NaNiO_2_ and NaTiO_2_, can be served as cathode and anode materials in SIBs, respectively. However, the poor structural stability of these materials derived from the complex phase transformation during their desodiation and sodiation makes their commercial applications still on the rock. Recent studies showed that the comprehensive electrochemical performance of these composites can be well improved in a solid‐solution state.^62^, ^63^ Considering the similar ionic radii of nickel and titanium, and also their unique double redox with a potential difference of 2.8 V, Ni^4+^/Ni^2+^ (3.5 V) and Ti^4+^/Ti^3+^(0.7 V), Zhou's group designed a O3‐type Na_0.8_Ni_0.4_Ti_0.6_O_2_‐based symmetric SIB (see **Figure** **3**).^64^ This cell exhibited a high average voltage of 2.8 V, a reversible discharge capacity of 85 mAh g^–1^, 75% capacity retention after 150 cycles, and good rate capability. In addition, Cr^3+^ and Ti^4+^, with the similar ionic radii and different redox potentials, were employed in a P2‐Na*_x_*[M1,M2]O_2_‐layered oxide system, leading to a completely Na^+^ vacancy‐disordered P2‐Na_0.6_[Cr_0.6_Ti_0.4_]O_2_ composite.^65^ Furthermore, Zhou's group developed a symmetric SIB system based on this P2‐Na_0.6_[Cr_0.6_Ti_0.4_]O_2_ electrode. 75% of the initial capacity at 12C rate could be retained in this cell.^65^ Although the research history of using titanate‐based symmetric electrodes in SIBs is slightly shorter than that of LIBs, a broader and deeper study has been carried out in SIBs. More importantly, a high specific capacity and excellent cycling stability can be found in the titanate salts‐based symmetric SIBs.

#### 2.2.3 Vanadate Salts‐Based Symmetric SIBs

Sodium vanadium oxides have also attracted considerable attention as electrodes in energy‐storage systems.^66^, ^67^ Layered NaV*_x_*O*_y_* materials, such as NaV_6_O_15_ and NaV_3_O_8_, showed the promising specific capacity in SIBs. Moreover, benefitting from the different oxidation states of vanadium, these materials can also be employed in a symmetric full cell. Srinivasan's group found that the pure Na_2.55_V_6_O_16_⋅0.6 H_2_O showed a high capacity and outstanding stability at high current rates up to 800 mA g^−1^ in the normal sodium half cells.^68^ Furthermore, a high discharge capacity of about 140 mAh g^−1^ was achieved in the Na_2.55_V_6_O_16_‐based symmetric SIBs with a cathode/anode mass ratio of around 11.

#### 2.2.4 Conductive Polymers‐Based SIBs

The research development of new kinds of sustainable and green electrodes is necessary for safer and less expensive SIBs production. The organic electric active materials are always endowed with potential advantages such as structural diversity and flexibility, molecular‐level controllability, eco‐efficient process ability and resource renewability. Therefore, developing organic compounds with multifunctional groups as electrode materials for rechargeable SIBs is very important.

**Figure 4:**
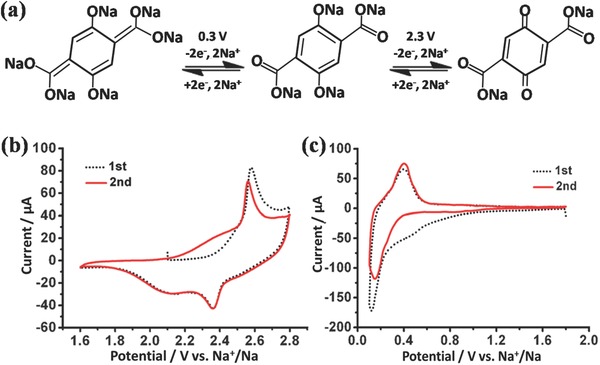
a) Electrochemical redox reaction mechanism of Na^+^ ions with Na_4_C_8_H_2_O_6_/Na_6_C_8_H_2_O_6_ and Na_2_C_8_H_2_O_6_/Na_4_C_8_H_2_O_6_ at potentials of 0.3 V and 2.3 V, respectively; CV curves of Na_4_C_8_H_2_O_6_/Na half cells at a scan rate of 0.1 mV s^−1^ in the voltage range of 1.6–2.8 V vs Na^+^/Na (b), and 1.8–0.1 V vs Na^+^/Na (c). Reproduced with permission.^69^

Recently, Chen's group reported the electrochemical performance of a novel organic tetrasodium salt of 2, 5‐dihydroxyterephthalic acid (Na_4_DHTPA) with enolate and carboxylate groups as the symmetric electrodes in SIBs.^69^ It was demonstrated that two reversible two‐Na^+^‐ion electrochemical reactions occur with redox couples of Na_2_C_8_H_2_O_6_/Na_4_C_8_H_2_O_6_ (see **Figure** **4**a) as the positive electrode at 2.3 V (see Figure 4b) and Na_4_C_8_H_2_O_6_/Na_6_C_8_H_2_O_6_ as the negative electrode at 0.3 V (see Figure 4c), respectively. Therefore, an all organic rocking‐chair symmetric SIB with an average output voltage of 1.8 V was fabricated. This result should shed light on the design and application of organic materials for room‐temperature rechargeable SIBs.

Compared with phosphate or titanate‐based symmetric electrodes, it is expected that the organic conducting polymers have some obvious advantages, such as lower price, elemental abundance, and higher specific capacity. We believe that these advantages will make organic molecules ideal candidates for rechargeable and safe symmetric electrode materials in SIBs in the future. However, the development of organic molecules in this field is slow at present, due to the issues of instability. Therefore, it is desirable to develop high‐performance symmetric electrodes made of organic molecules.

### 2.3 Symmetric Electrochemical Capacitors

A great deal of attention has been focused on electrochemical capacitors (ECs) because of their longer cycle life, lower maintenance cost, quicker rate‐response in comparison to traditional batteries and significantly high power density, leading to their broad commercial applications.^70^, ^71^ Theoretically, electrical energy can be stored in the ECs at the electrode/electrolyte interface via an electrostatic interaction of charges.^72^ Generally, supercapacitors are classified into two types: electric double‐layered capacitors (EDLCs) and Faradaic pseudo capacitors.^73^, ^74^ Carbon‐based materials with the repaid current–voltage *(I*–*V)* response and rectangle cyclic voltammetry (CV) shape have been so far the representative electrode material of EDLCs. For pseudo capacitors, RuO_2_, conducting polymers, and transition‐mental oxides are typical electrode materials for ECs.

**Figure 5:**
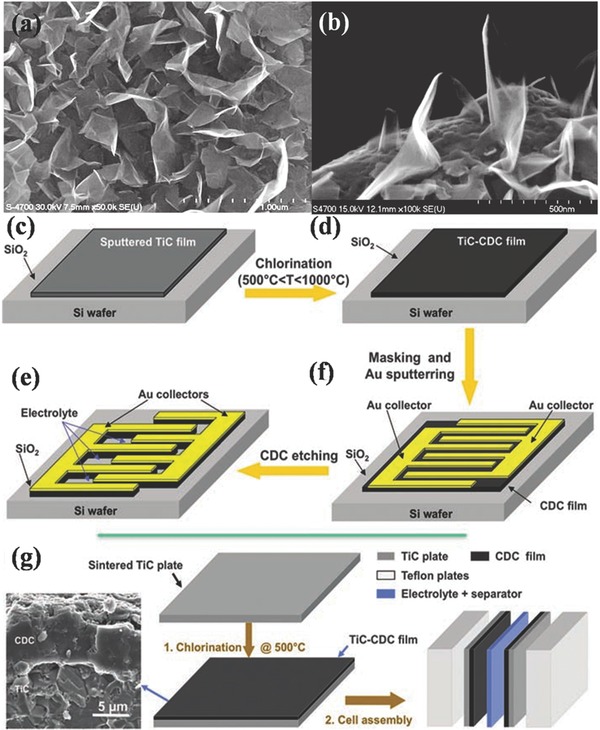
a) Plan SEM image of coated Ni electrode, b) SEM image of a coated fiber, showing plan and shallow‐angle views. Reproduced with permission.^76^ Copyright 2010, Science; c–f) Schematic of the fabrication of a micro‐supercapacitor integrated onto a silicon chip based on the bulk carbide‐derived carbon (CDC) film process. g) CDC synthesis and electrochemical test cell preparation schematic. c–f) Reproduced with permission.^77^ Copyright 2010, Science.

Generally, in a symmetric EDLC system, both the positive and negative electrodes in EDLCs are identical activated carbon (AC) materials. In order to introduce the porosity into AC materials to give a larger surface area and more active sites, KOH is often used to react with AC through thermal treatment at an inert atmosphere (e.g., Ar and N_2_). As a result, a large numbers of carbon atoms could be “corroded” off during the thermal KOH‐assisted treatment, leading to the porous architecture within AC, but the process is uneconomic for large‐scale production for EDLCs. Also, the limited energy density of AC‐based EDLCs cannot satisfy the increasing demand in the energy‐storage devices. Sumio Iijima's group^75^ outlined a general and rational strategy to fabricate an aligned high‐densely packed single‐walled carbon‐nanotube (SWNT) solid material by using the zipping effect of liquids to draw nanotubes together. The capacitance of this SWNT solid EDLC was around 80 F g^−1^. B. C. Holloway' group^76^ demonstrated efficient filtering of 120‐Hertz current with EDLCs with electrodes made from vertically oriented graphene nanosheets grown directly on metal current collectors (**Figure** **5**a,b). Graphene nanosheets have a preponderance of exposed edge planes that greatly increases charge storage. Capacitors constructed with these electrodes could be smaller than the low‐voltage aluminum electrolyte capacitors that are typically used in electronic devices. By etching supercapacitor electrodes into conductive titanium carbide substrates, Yury Gogotsi's group demonstrate that monolithic carbon films lead to a volumetric capacity exceeding that of micro‐ and macroscale supercapacitors reported thus far (see Figure 5c–g).^77^

Recently, a number of excellent reviews and reports on the design, preparation, modification, characterization, properties, engineering, and applications of carbon‐based materials for symmetry‐structured EDLCs have been published.^78^, ^79^, ^80^, ^81^, ^82^, ^83^, ^84^, ^85^, ^86^, ^87^

## 3 Summary and Open Questions

We have summarized the recent advances in different symmetric energy storage systems, such as LIBs, SIBs and ECs. Symmetric electrodes have been demonstrated to be promising for the emerging energy‐related applications. Great efforts have been devoted to investigating new symmetric electrode materials in recent years, taking advantages of their high safety, low‐cost and scalable ability, and overcoming its low energy densities by introduction of more electrochemical active positions and nanosized structures. Despite many significant achievements, various challenges still remain and need to be addressed to prepare high‐performance symmetric energy systems.

Compared with the LIBs and SIBs, the researchers' attention about the symmetric energy storage technique was first attracted by ECs. Carbon‐based and polymer or transition mental oxides were first employed as the symmetric electrodes in the EDLC. As for the symmetric LIBs, compared with the titanate‐ and vanadate‐based salts, phosphate salts are more promising when employed as symmetric electrodes. NASICON‐type LVP has a high theoretical capacity of 197 mAh g^−1^ and excellent conductivity. In addition, the reactions of Li^+^ with LVP proceed at around 4.1 and 1.7 V, resulting in a large output voltage. Moreover, the subsequent doping of other elements (F, Co, Fe, Ti and Ni) in LVP can further improve its electrochemical performance. Such properties make the monoclinic LVP attractive as symmetric electrodes in LIBs. Symmetric SIBs are the newly emerged energy storage technique during the past six years. Different from LIBs, the scalable production cost of SIBs is much lower than that of LIBs, due to the abundant reserve of sodium on the earth. Therefore, from a view of massive production in terms of the cost and available possibilities of scalable production, the symmetric LIBs may be not the optimum choice and symmetric SIBs are more promising. Compared with other symmetric electrodes, such as phosphate and titanate salts, conducting polymers should be our best choice, because of their structural diversity and flexibility, molecular level controllability, eco‐efficient process ability and resource renewability. Furthermore, by increasing the active positions in the organic molecule, such as carbonyl group and N atoms, the specific capacity can be significantly increased, which makes conductive polymer‐based symmetric SIBs are attracting more and more researchers' attention.

Among all above mentioned different symmetric energy storage systems so far, the symmetric SIBs seem to be most promising, because the rechargeable batteries have already massively produced and cost‐effectively controlled. Taking LIB as an example, it is being widely applied in various fields and already covered almost all aspects of our city life. Unfortunately, the limit reserve of Li on the earth leads to an increasing cost in a massive production. Therefore, symmetric SIBs would be a selling point in the future. Nevertheless, some further investigations are definitely needed. There are some considerations in this context: (1) a better understanding about the mechanism of the electrochemical reaction between Na^+^ and the active sites in the symmetric electrodes would be highly desirable, because a certain amount Na^+^ cannot keep their reversible active during the charge/discharge reactions, so this is very important to improve the efficiency and reduce the materials waste; (2) although the symmetric SIB, especially the polymer‐based ones, are more promising compared with other systems, the low cost and the availability of mass production are always the final targets for the commercialization; (3) different from the asymmetric systems, some symmetric electrodes need to be prelithiated/predelithiated treatments before cell fabrication, enabling the electrodes in lithiated/delithiated states. However, this may bring about the assemble process more complex; and (4) furthermore, the theoretical specific capacities of symmetric electrodes are generally poor in contrast to asymmetric ones due to the narrow working potentials, thus limiting their particle applications. Therefore, how to further reduce the production cost and simplify the synthesis technology is still the primary consideration.
